# Antimicrobial Activity of *Bacillus* Cyclic Lipopeptides and Their Role in the Host Adaptive Response to Changes in Environmental Conditions

**DOI:** 10.3390/ijms26010336

**Published:** 2025-01-02

**Authors:** Natalia Markelova, Anastasia Chumak

**Affiliations:** Gause Institute of New Antibiotics, ul. Bolshaya Pirogovskaya, 11, Moscow 119021, Russia; nastya-chymak@mail.ru

**Keywords:** cyclic lipopeptides, genus *Bacillus*, antimicrobial activity, cell differentiation, adaptive response

## Abstract

*Bacillus* cyclic lipopeptides (CLP), part of the three main families—surfactins, iturins, and fengycins—are secondary metabolites with a unique chemical structure that includes both peptide and lipid components. Being amphiphilic compounds, CLPs exhibit antimicrobial activity in vitro, damaging the membranes of microorganisms. However, the concentrations of CLPs used in vitro are difficult to achieve in natural conditions. Therefore, in a natural environment, alternative mechanisms of antimicrobial action by CLPs are more likely, such as inducing apoptosis in fungal cells, preventing microbial adhesion to the substrate, and promoting the death of phytopathogens by stimulating plant immune responses. In addition, CLPs in low concentrations act as signaling molecules of *Bacillus*’s own metabolism, and when environmental conditions change, they form an adaptive response of the host bacterium. Namely, they trigger the differentiation of the bacterial population into various specialized cell types: competent cells, flagellated cells, matrix producers, and spores. In this review, we have summarized the current understanding of the antimicrobial action of *Bacillus* CLPs under both experimental and natural conditions. We have also shown the relationship between some regulatory pathways involved in CLP biosynthesis and bacterial cell differentiation, as well as the role of CLPs as signaling molecules that determine changes in the physiological state of *Bacillus* subpopulations in response to shifts in environmental conditions.

## 1. Introduction

Most members of the genus *Bacillus* are saprophytes. The habitat of *Bacillus* is all types of soil with different acidity and temperature conditions, fresh and sea water, bottom sediments, and thermal springs; they are also found in plants and animals. The widespread distribution of *Bacillus* in the environment is facilitated by the endospores they form, which are resistant to physical and chemical agents. In addition, bacteria of the genus *Bacillus* are characterized by the production of various secondary metabolites, some of which inhibit the growth and development of competing microorganisms, providing the producers of these compounds with an advantage in colonizing new habitats [1]. Such compounds also include cyclic lipopeptides (CLPs), known for their antagonistic role in relation to a wide range of microorganisms, especially phytopathogens. Therefore, many members of *Bacillus* are closely associated with plants, and such species as *B. amyloliquefaciens*, *B. endophyticus*, *B. licheniformis*, *B. megaterium*, *B. pumilus*, *B. subtilis* were isolated from their internal tissues, where, in addition to protection against pathogens, they perform a symbiotic function of stimulating plant growth [2].

Pathogenic fungi not only cause plant diseases that result in significant crop losses but also pose risks to food safety. One approach to combating fungal plant pathogens is the use of biofungicides, which include *Bacillus* species producing CLPs [3]. The most promising species that can be used as biofungicides are *B. amyloliquefaciens*, *B. velezensis*, *B. subtilis*, *B. nakamurai*, *B. siamensis*, *Bacillus licheniformis* [4].

*Bacillus* CLPs are represented by three main families of nonribosomally synthesized peptide derivatives, surfactin, iturin, and fengycin, and lesser-known compounds, which include curstacins and lociillomycins [5,6]. Many *Bacillus* strains are capable of producing more than one compound and in various combinations, which increases their effectiveness against phytopathogenic fungi. *B. amyloliquefaciens MR4* harbors genes for 13 secondary metabolites, including surfactin and fengycin. The strain suppressed the pathogens of cotton wilt and verticillium wilt with an inhibition efficiency of over 50% [7]. *B. velezensis* R22 contains 10 gene clusters of secondary metabolites associated with antimicrobial activity, including fengycin and surfactin. This strain exhibited the highest antifungal activity against *B. cinerea* and *P. infestans* [4]. Most *B. velezensis* strains can synthesize all three types of lipopeptides, namely surfactin, fengycin, and iturin [8,9,10,11]. Some strains have proven to be overproducers of CLPs; for example, *B. velezensis* UTB96 can produce higher amounts of all three CLPs compared to the commercial strain *B. velezensis* FZB42 [12]. Other strains, such as *B. velezensis* SDTB038, have exhibited greater efficacy than chemical fungicides [13]. Biofungicides contain microorganisms that are active against their target fungal pathogens. Unlike toxic chemical fungicides, they are safe for plants, insects, and animals, demonstrate effective control of fungal plant pathogens, and may surpass chemical fungicides in effectiveness.

CLPs are a part of a larger group of bioactive lipopeptides produced not only by *Bacillus* but also by related species of the genus *Paenibacillus*, some of which were previously classified as *Bacillus* species [14]. In general, this group of compounds has not previously attracted attention as potential antibiotics due to the presence of systemic toxicity in these compounds. Only with the widespread use of multidrug-resistant bacteria and the lack of effective drugs against them were lipopeptides again considered as promising antibacterial drugs; notably, the FDA (US Food and Drug Administration) approved the clinical use of *polymyxin B*, a cyclic lipopeptide produced by *Paenibacillus polymyxa* [15,16]. *Bacillus* CLPs exhibit varying levels of cytotoxicity and differ in biological activity. Iturins demonstrate antifungal and antitumor activity but also significant hemolytic activity. Fengycins also display antifungal activity and limited cytotoxicity. Surfactins are less hemolytic than iturins, but more so than fengycins [5,16]. The antibacterial activity of this group of compounds is limited, which is associated with differences in the structure of prokaryotic and eukaryotic membranes. Prokaryotic membranes are dominated by charged lipids, and the proportion of negatively charged lipids influences the selectivity of cationic antimicrobial peptide binding to these membranes. The *Bacillus* CLPs group is classified as non-cationic lipopeptides, and their predominant biological activity targets eukaryotic cells, whose membranes predominantly contain uncharged lipids. Therefore, fungi and enveloped viruses, which carry host membranes, are more frequently targeted by the antimicrobial action of CLPs [17]. In addition to their antifungal activity, CLPs also target mammalian cells. They exert a cytotoxic effect on cancer cells, inhibiting the proliferation and survival of tumor cells, and therefore have potential as antitumor agents [16]. Surfactin, for example, is active against several cancer cell lines associated with breast, colon, and blood diseases [18].

CLPs have some other potential pharmaceutical and biomedical applications. CLPs act as anti-inflammatory agents, promoting wound healing. Iturin enhances tissue regeneration by stimulating angiogenesis and has a local antimicrobial effect [19]. Surfactin also promotes wound healing by altering the gene expression of proteins involved in this process and inhibits scar tissue formation [20]. CLPs are natural biosurfactants and can be used as solubilizers, stabilizers, and emulsifiers of drugs when delivering them to therapeutic targets. Iturin molecules are capable of spontaneously forming micelles or vesicles with encapsulated hydrophobic compounds, which can be used for the transport of hydrophobic drugs [19]. Surfactin was used to stabilize nanoparticles against aggregation, sedimentation, and other destabilization phenomena used for transdermal delivery of substances [21].

Today, the main families of CLPs from bacteria of the genus *Bacillus*—iturins, surfactins, and fengycins—are well known; their chemical structures, biosynthesis gene clusters, and mechanisms of antagonistic action against other microorganisms have been characterized. Most of the results achieved in the study of CLPs from *Bacillus* were associated with efforts to discover CLPs useful for medicine, pharmacy, agriculture, and the biotechnological processes involved in their production. At the same time, CLPs, like other secondary metabolites of *Bacillus*, perform several essential functions for bacteria in their natural habitat. In this context, the antimicrobial action of CLPs should be considered as a natural function in microbial antagonism. No less important is the role of CLPs in the metabolism of producer bacteria, where they function as signaling molecules for coordinated growth and differentiation, thus implementing protective mechanisms in response to the negative impact of other microorganisms and environmental factors.

The aim of this review is to summarize our current understanding of the antimicrobial action of *Bacillus* CLPs, their role in antagonistic relationships between bacteria in natural habitats, and the involvement of CLPs in the metabolism of bacteria producing these compounds, which promotes bacterial adaptation to environmental changes.

## 2. CLP-Producing *Bacillus* and Methods of Their Detection

Whole genome sequencing (WGS) of *Bacillus* and analysis of multiple genes have facilitated comparative genomic studies and improved the clarification of species affiliation within the genus *Bacillus*. Many strains of this genus were previously identified, including through 16S rRNA gene analysis, as *B. subtilis* and *B. amyloliquefaciens*, but were later reclassified as belonging to other species or even genera. Some strains of *B. subtilis* and *B. amyloliquefaciens* were reclassified as *B. velezensis* [22,23]. In general, the *Bacillus* genome, using *B. velezensis* as an example, is about 4 million bp. in length with a G + C content of about 46% and contains roughly 4000 protein-coding genes, tRNA and rRNA [4,11]. In silico genome-based methods of studying the whole-genome phylogeny and functional annotation of the pathways of *Bacillus* main genes revealed common patterns for these bacteria. As a result, the predominance of pathways involved in key cellular processes was discovered, including DNA replication, fatty acids biosynthesis, folate biosynthesis, nitrogen metabolism, purine and pyrimidine metabolism, protein export, ribosome formation, and the presence of ATP-binding cassette (ABC) transporters. ABCs are able to bind and remove substances against a concentration gradient by hydrolyzing ATP, which is important for neutralizing toxic compounds in the environment and providing autoimmunity to chemical-producing bacteria. The most prevalent classes of *Bacillus* biosynthetic genes (≥2% of total BGC diversity) included CLPs [24]. CLP genes are organized into operons and share common regulatory pathways. The SrfA operon is part of the srfA operon-sfp gene cluster, which is 30.5 kb in length. Surfactin synthetase is encoded by four open reading frames (ORFs) from srfAORF1 to srfA0RF4, encoding four enzymatic subunits, SrfAA, SrfAB, SrfAC, and SrfAD. The region between the srfA operon and the sfp gene contains four ORFs that do not affect surfactin production, and the sfp gene is responsible for the docking sites of specific amino acids in surfactin synthetase [25]. The iturin A operon is more than 38 kb in length and consists of four ORFs: ituD, which encodes malonyl-CoA transacylase, and ituA, ituB, and ituC, which encode amino acid-activating modules and subunits of bacillomycin D synthetase [26]. The operon encoding fengycin synthetase is approximately 38 kb in length and consists of five ORFs: fenC, fenD, fenE, fenA, and fenB [27]. The main regulators of CLP synthesis are transcription factors (sigmaH, Spo0A, AbrB), two-component systems (degU, degQ), and quorum-sensing systems (ComA, PhrC) [25,27].

Today, the main producers of CLPs form the phylogenetic group *B. subtilis*, which includes the species *B. amyloliquefaciens*, *B. licheniformis*, *B. pumilus*, and the type species *B. subtilis* [28]. Table 1 presents various *Bacillus* species and the CLP variants they produce.

Despite the fact that bacterial whole-genome or metagenome sequencing is widely used in laboratories and DNA assembly techniques are becoming increasingly advanced, other methods of molecular genetic analysis of CLPs remain highly relevant. To identify CLPs and quantify the expression of CLP synthesis genes in *Bacillus* species, the quantitative polymerase chain reaction (PCR) with reverse transcription RT-qPCR is used. The method is based on the isolation of RNA from bacteria, performing a reverse transcription reaction, obtaining complementary DNA (cDNA), and amplification of the biosynthetic cluster genes of the target product. As a result, the presence of biosynthetic genes involved in the production of CLPs and the expression level of these genes are determined [81]. In some studies, PCR is used to detect genes of nonribosomal peptide synthetases, such as fengycin (fenA, fenA), surfactin (srfAA, srfAB, srfAC), lichenisin (lchAA), and to obtain nucleotide sequences of the 16S rRNA gene for further sequencing and determination of the genus, and less often, the species affiliation of *Bacillus* [4,13].

The rediscovery of already-known compounds under standard laboratory conditions and a significant number of silent biosynthetic gene clusters (BGCs) remains a major obstacle in the discovery of new *Bacillus* CLPs. Whole-genome sequencing of bacterial genomes and their bioinformatic analysis allow the identification of a variety of BGCs, including previously uncharacterized metabolites. Using bioinformatics tools such as PRISM or antiSMASH, it is possible to quickly predict BGCs and the chemical compounds encoded by them. Additionally, the IMG-ABC v5.0 and MIBiG 2.0 repositories of known compounds were developed for in silico analysis of new BGCs, enabling the assessment of homology in the BGCs under study [82,83]. A recent study of 6378 *Bacillus* genomes from 66 different species using the BiG-SCAPE and BiG-SLiCE computational systems revealed species specificity in the distribution of BGCs and showed that the NRPS class is the most abundant among BGCs [84]. It is worth noting that not only is the in silico prediction of CLP biosynthesis possible through bioinformatics methods, but the chemical synthesis of these compounds is also possible using an approach called synthetic-bioinformatic natural product (syn-BNP). This approach enables the total chemical synthesis of linear or cyclic peptide products based on the predicted structures derived from BGC sequences [82].

## 3. Structure and Mechanism of Action of Cyclic Lipopeptides

**Figure 1:**
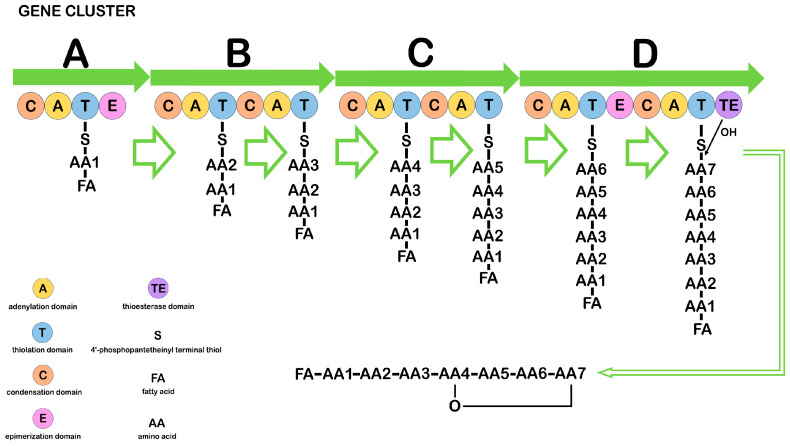
Diagram illustrating the structure and function of the nonribosomal peptide synthetase responsible for synthesizing CLPs in *Bacillus*. A, B, C, D—main biosynthesis genes. (Figure 1 was created using the images from Servier Medical Art https://smart.servier.com/, 8 December 2024.)

CLPs are synthesized by multienzyme complexes called nonribosomal peptide synthetases (NRPS) (Figure 1).

The minimal NRPS module consists of three catalytic domains, adenylation (A), thiolation (T), and condensation (C), and it represents the basic repeating unit of the multimodular NRPS. Nonribosomal peptide (NRP) synthesis occurs in several stages: the A-domain recognizes and activates a certain amino acid, forming an intermediate product aminoacyl–AMP, which controls the entry of the amino acid into the neighboring T-domain. Substrates of the A-domain can be not only proteinogenic amino acids but also non-proteinogenic ones, which determines the structural diversity of NRPs. The T-domain is post-translationally modified by a 4′-phosphopantetheinyl group that binds the invariant serine residue in the domain. The terminal thiol of the 4′-phosphopantetheinyl group interacts with the carboxyl group of aminoacyl–AMP on the A-domain to form intermediates. Thus, T-domains function as carriers and are known as peptidyl carrier proteins. The C-domain sequentially connects two amino acids by a peptide bond, one of which is derived from the previous module and the other from the current module. Three main domains—C, A, and T—within the modules can be supplemented by epimerase and methyltransferase domains, catalyzing amino acid modifications such as epimerization and N-methylation, respectively, which contributes to an increase in the structural complexity of NRPs. The release of peptides from NRPSs is usually catalyzed by the thioesterase domain through hydrolysis, aminolysis, or intramolecular cyclization [5,85,86,87,88,89]. The number of modules within the NRPS gene clusters responsible for the synthesis of different CLPs is not the same. The order and specificity of the modules determine the amino acid sequence for linear NRPSs such as surfactin, bacillomycin L, and fengycin. Other types of NRPSs may reuse a module or specific domains during NRP assembly. For example, the nonribosomal loc gene cluster, which is responsible for locillomycin synthesis, deviates from the linear mechanism. The cluster includes the locD, locA, locB, and locC genes and a hexamodular assembly line in which the middle three modules are used twice, and the first and last two modules are used only once [5,85]. The surfactin and fengycin clusters contain NRPS genes, and the iturin gene cluster is a hybrid of a polyketide synthase (PKS) and an NRPS. Genes containing PKS, such as ituA from the iturin A cluster or the mycA gene from the mycosubtilin cluster, contain three functional domains with fatty acid synthetase, amino acid transferase, and peptide synthetase [5,88,89].

**Figure 2:**
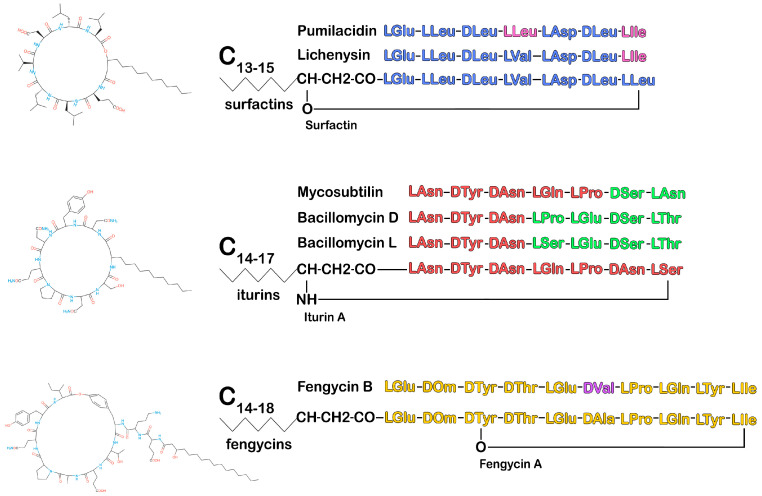
Chemical structure diagram of the *Bacillus* CLPs: surfactins, fengycins, and iturins. Amino acids in the peptide chain that differ from those in the peptide chain of the main representative of the CLP family are highlighted in a different color. (Figure 2 was created using the images from Servier Medical Art https://smart.servier.com/, 8 December 2024.)

Unlike linear lipopeptides, surfactins, iturins, and fengycins have a cyclic structure formed by 7–10 amino acids, including 2–4 D-amino acids and beta-hydroxy fatty acid or beta-amino fatty acid with 13–19 carbon atoms. Each family produces variants with the same peptide length but different amino acid sequences. Each variant includes homologs that differ in the length and branching of fatty acid chains, which leads to significant structural heterogeneity and determines the properties of CLPs (Figure 2) [90,91].

Although all CLPs have a cyclic three-dimensional structure, this does not always reflect similarity in their biological activity. The structures of the acyl side chains and the different peptides within CLPs, some of which include unusual (non-coded or non-proteinogenic) amino acids, significantly affect the biological activity of CLPs both in vitro and in vivo. Cleavage of the CLP cyclic structure results in the loss of their biological activity. In nature, the chemical stability of CLPs is maintained due to their insensitivity or weak sensitivity to plant or animal peptidases [92]. However, degradation of CLPs occurs, and a novel role for CLPs in interspecies interactions has even been suggested. *Streptomyces venezuelae* has been shown to enzymatically degrade surfactin, iturin, and fengycin when interacting with *B. velezensis* in vitro and in planta, which favors the maintenance of nitrogen metabolism in the rhizosphere microbial community [93]. In terms of CLPs’ potential as antimicrobials, the presence of unusual amino acids and a mixture of D- and L-amino acids in the substance increase the stability of CLPs against proteolytic enzymes of target organisms and mammalian proteases [16].

The biological activity of CLPs is closely related to their chemical characteristics. CLPs are amphiphilic membrane-active peptides with pronounced antimicrobial properties, the antibacterial activity of which decreases, and the antifungal activity increases with the increase in the length of the carbon chain of fatty acids. Presumably, all lipopeptide compounds with a chain of 10–16 carbon atoms in the aliphatic chain tend to form oligomers in the plasma membrane and form pores deeply embedded in it, which increases the fungicidal activity of lipopeptides [94,95,96].

Membrane disruption is the primary action of many lipopeptides, including CLPs, where resistance development is impeded due to complex membrane reorganization by target cells [16]. Although the fact of membrane disruption has been confirmed, the mechanism by which CLPs interact with membranes is not fully understood. The immediate targets to which CLPs attach on the membrane surface likely depend on the charge, composition of membrane phospholipids, and other membrane components. Bacterial membranes are typically negatively charged due to the presence of phosphatidylglycerol and cardiolipin, and Gram-negative bacteria contain more phosphatidylethanolamine than Gram-positive bacteria, so interactions with charged membranes are likely caused by electrostatic forces. Eukaryotic membranes are dominated by uncharged lipids, where hydrophobic interactions between CLP and membrane components are more likely to occur [17].

**Figure 3:**
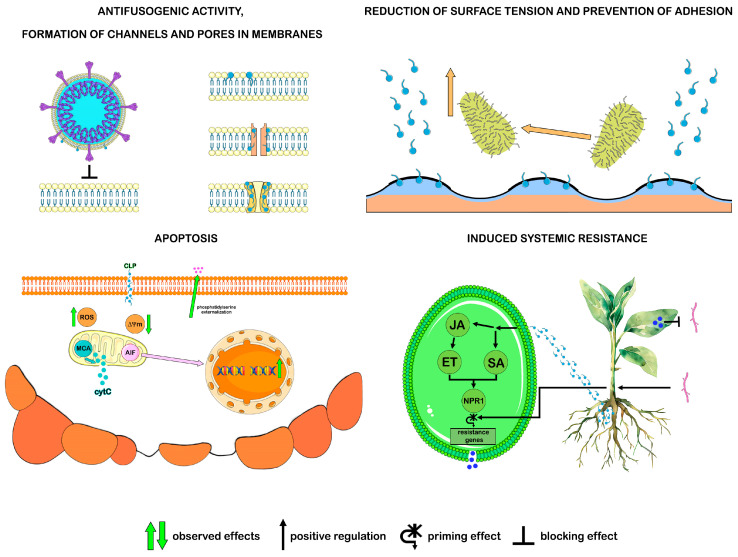
Antimicrobial activity of surfactins, fengycins, and iturins. ISR: CLP induces phytohormone signaling pathways by activating the NPR1 protein. Pathogen infection removes the priming effect and activates the transcription of defense response genes. Apoptosis: CLP causes apoptotic changes in fungal cells: phosphatidylserine externalization, ROS accumulation, decreased mitochondrial membrane potential decrease, and DNA fragmentation. There are different ways of implementing this process in fungi: mitochondrial metacaspase (MCA) facilitates the release of cytochrome C into the cytoplasm, triggering the caspase-dependent apoptosis pathway; apoptosis-inducing factor (AIF) activates the caspase-independent apoptosis pathway. (Figure 3 was created using the images from Servier Medical Art https://smart.servier.com/, 8 December 2024).

In vitro studies have revealed the antimicrobial activity of CLPs against a wide range of microorganisms, including viruses, bacteria, and fungi, while their direct inhibition caused by CLPs may be only one of the ecological roles of these compounds in the natural environment. With the properties of biosurfactants, CLPs participate in antibiosis, preventing the adhesion of microorganisms to the substrate and the formation of biofilms, also destroying the latter. In subinhibitory concentrations, CLPs have a specific effect on the activity of enzymes, proteins, and nucleic acids of microbes, which leads to the suppression of their growth or death. Thus, without causing a significant toxic effect, CLPs trigger apoptosis in fungal cells by interfering with their cell cycle [97,98,99]. In addition to direct antagonistic interaction with microorganisms, CLPs are able to stimulate plant defense mechanisms through the phenomenon of induced systemic resistance (ISR), which eliminates the need for direct interaction between the pathogen and the resistance-inducing agent. The plant defense response associated with ISR is realized through the induction of phytohormone signaling pathways, such as the jasmonic acid (JA) and ethylene (ET) pathways, where CLPs can act as their inducers. The ISR response depends on the activation of the positive regulatory protein NPR1, which is common to phytohormone signaling pathways. Activation of NPR1 leads to a priming effect that is not accompanied by transcription of genes associated with plant defense. After the onset of pathogen infection, the priming effect is removed, and transcription of defense response genes occurs (Figure 3) [42,100,101].

By secreting CLPs, bacilli not only participate in antagonistic interactions with other microorganisms but also use CLPs in their own metabolism and interactions with the environment. The amphiphilic nature of CLPs and their low concentrations in natural conditions determine the functions of CLPs, such as reducing or enhancing motility and biofilm formation [85,92,93]. These functions may be associated with the action of CLPs, both as surfactants and as signaling molecules. Experimental suppression of the NRPS genes responsible for CLP synthesis in bacilli resulted in a change in their motility and a decreased ability to form biofilms. Acting as signals, CLP can regulate the multicellular behavior of bacteria, such as swarming motility and biofilm formation. It has been shown that blocking CLP synthesis affects the differentiation of bacterial cells into specialized flagellate forms. Acting as a surfactant, CLP reduces the surface tension on biotic and abiotic surfaces, changing their viscosity. Changes in surface properties also affect biofilm formation and motility at the stage of attachment to the aforementioned surface. It has been suggested that this stage depends on the surface charge of the bacteria and substrate, as well as the charge and hydrophobicity of CLP. Thus, by altering the swarming motility of host bacteria and influencing their biofilm formation, CLPs facilitate the colonization of favorable habitats by these bacteria, help evade antibacterial substances, and protect against other adverse environmental factors [6,97,98].

## 4. Surfactins

Surfactin was isolated and identified in the culture medium of *B. subtilis* in 1968 as the first biosurfactant [102]. Surfactin, lichenisin, and pumilacidin are the main members of another family of CLPs produced by *Bacillus*—surfactins. The peptide component of surfactin consists of seven amino acids arranged in a chiral central sequence (LLDLLDL), which is cyclized with a β-hydroxy fatty acid to form a lactone structure. The carbon chain of the β-hydroxy fatty acid contains 13 to 15 carbon atoms (Figure 2). Surfactin homologs vary in their unique combination of amino acids, the length of the fatty acid chain, and, less commonly, their structural conformation [91,100,103]. Surfactin biosynthesis is most commonly observed in *B. subtilis*, *B. velezensis*, *B. amyloliquefaciens*, and *B. pumilus* [104].

Surfactin is a typical compound of the family that has been studied in detail, so it seems appropriate to consider this compound as an example. Surfactin’s activity against a wide range of microorganisms primarily stems from its interaction with the lipid components of bacterial and fungal membranes, as well as the lipid envelopes of viruses, ultimately destabilizing these structures. The mechanism surfactin interaction with the membrane depends on its concentration. At high concentrations, the substance disrupts the integrity of cell membranes in a manner similar to the action of a detergent based on the solubilization of the liquid phospholipid phase of the membrane. Above the critical micelle concentration, where stable micelles form, the concentration of surfactin molecules leads to the disintegration of bilayer membrane structures and subsequent cell lysis [105,106]. In addition to decay, surfactin forms channels and pores in cell membranes. When amphiphilic surfactin molecules are incorporated into the lipid bilayer, they can initiate the self-assembly of multimers that form channels [107]. The inclusion of aggregated peptide helices into the membrane increases its instability, leading to the formation of pore-like structures (Figure 3) [108]. The presence of channels and pores in the membrane changes its permeability, causing an imbalance in osmotic pressure inside and outside the membrane, releasing vital ions from the cell and ultimately leading to the death of microorganisms. The antimicrobial effect of surfactin may also target other cellular targets, such as RNA, DNA, or proteins, disrupting microbial metabolism and development [17].

Surfactin exerts not only a direct antimicrobial effect on microorganisms but also an indirect effect by altering the properties of surfaces they attach to. Its antiadhesive action stems from its ability to adsorb onto solid surfaces and reduce their hydrophobicity, thereby weakening hydrophobic interactions with microbes. In addition, this effect can be amplified by electrostatic repulsion between microorganisms and surfactin molecules. These interactions inhibit microbial attachment to substrates and prevent biofilm formation [109].

The antibacterial effect of surfactin in most studies is associated with the destruction of membranes or with antibiofilm activity. For example, disruption of the cell membrane integrity in *P. acnes* occurs when the long-chain fatty acid tail of surfactin integrates into the phospholipid bilayer, leading to solubilization of the liquid phospholipid phase and pore formation. Membrane damage in *P. acnes* was confirmed by the penetration of propidium iodide, a dye that cannot penetrate an intact plasma membrane [110]. Although surfactin is recognized as a compound with high antimicrobial potential, it is important to note that its inhibitory effect on bacteria has been observed only at relatively high concentrations of 50–400 μg/mL [32]. Such concentrations are much higher than those typically found in nature. However, as a potent biosurfactant, surfactin can effectively reduce surface tension at phase boundaries, even at very low concentrations (Figure 3). Therefore, under natural conditions, surfactin’s antibiofilm activity against bacteria is more likely to occur than its direct effect on bacterial cells [25]. Studies have shown that surfactin reduces the adhesion of *S. aureus* and destroys its biofilms on surfaces by decreasing the production of biofilm matrix polysaccharides and suppressing the expression of the *icaA* and *icaD* genes required for biofilm formation [111]. It also prevented the adhesion and formation of biofilms by food-pathogenic bacteria *Listeria monocytogenes*, *Enterobacter sakazakii*, and *Salmonella enteritidis* and demonstrated antimicrobial and antibiofilm activity against Legionella pneumophila [33,112]. Surfactin-15 F disrupted the structure of the pre-formed *S. epidermidis* biofilm and reduced its viability [113].

Surfactin exhibits antiviral activity. The antiviral effect of surfactin is, in some cases, attributed to its ability to penetrate the lipid layers of the viral envelope, leading to their solubilization and permeabilization, which results in the disintegration of viral particles. This mechanism can inactivate various enveloped viruses, particularly herpes and retroviruses [34]. Another potential antiviral mechanism of surfactin, as a membrane fusion inhibitor, was analyzed using the porcine epidemic diarrhea virus (PEDV) and the transmissible gastroenteritis virus (TGEV). It has been shown that surfactin incorporation into the viral envelope lipids reduced the likelihood of viral fusion with host cell membranes, thereby suppressing infection of these cells by enveloped viruses (Figure 3) [114]. The bioinformatics method of molecular docking was used to determine the binding energy of lipopeptides to the enzyme nsp12, an RNA-dependent RNA polymerase responsible for virus replication. Surfactins can potentially bind to SARS-CoV-2 nsp12 as effectively as sofosbuvir triphosphate (STP), a drug approved by the US Food and Drug Administration (FDA) [115]. Additionally, in silico analysis of the binding energy of various lipopeptides to the spike glycoprotein (S protein) demonstrates surfactin’s ability to inhibit the binding of virions to host cell receptors [116]. In a cellular infection model using Vero E6 cells, the anticoronavirus activity of surfactin against SARS-CoV-2 was confirmed through its direct destructive effect on the virion. It was possible to block viral replication in the experiment only by pre-mixing the compound and the virus [117].

The antifungal action of surfactin has been described for the genera *Fusarium*, *Trichoderma Aspergillus*, and *Penicillium* [32,118]. Surfactin interacts with phospholipids of fungal cell membranes, but the subsequent antifungal activity of the compound depends on its concentration. A study of surfactin from *B. amyloliquefaciens* WH1, known as WH1fungin, demonstrated that at high concentrations, it formed pores in the cell membrane, while at low concentrations, apoptosis was observed (Figure 3). Fungin WH1 bound to ATPase on the mitochondrial membrane in fungal cells and reduced its activity. The subsequent release of cytochrome C from the mitochondria and its interaction with caspase 9 induced apoptosis via the intracellular pathway. At the same time, the detected activity of caspase 8 in the same cells may indicate apoptosis via the extracellular pathway. Given that pore formation requires high concentrations of surfactin, which are difficult to achieve in natural environments, this mechanism may not be the primary one in competitive interactions with other microorganisms. Therefore, the suppression of fungal growth by *Bacillus* species through apoptosis induction may be a more natural function of surfactin compared to pore formation in fungal cell membranes [119].

Indeed, the antimicrobial action of surfactin, as a secondary metabolite released into the environment surrounding the producer bacterium, provides it with a competitive advantage in its ecological niche. On the other hand, interactions between microorganisms also suggest protection of the producer bacterial population from antimicrobial substances produced by other types of microorganisms. Studies on the function of surfactin in the producer bacterium cells revealed that it induces the transcription of biofilm-forming genes, acting as a signal molecule. In addition to matrix production by some cells, surfactin triggers the differentiation of *Bacillus* cells into subpopulations responsible for spore formation, motility, and cannibalism, while surfactin-producing cells are resistant to its effects.

**Figure 4:**
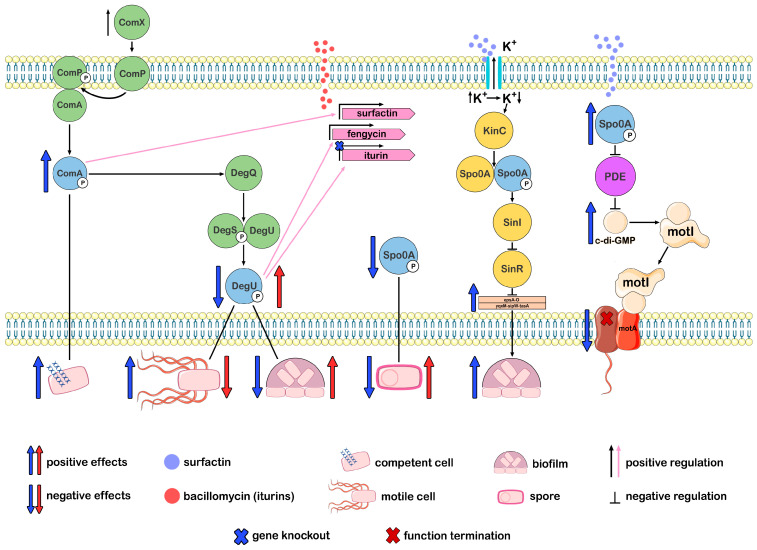
Differentiation of *Bacillus* cell subpopulations associated with the impact of CLPs and the key regulators of this process: ComA∼P, DegU∼P, and Spo0A∼P.

ComA and Spo0A proteins serve as the primary regulators of the surfactin synthesis process. Regulation occurs via activation of the transcriptional regulatory protein ComA, a product of one of the comQXPA genes, by its attachment to the promoter region of the srfA operon and initiation of surfactin synthetase transcription, consisting of four enzymatic modules [25]. Subpopulations of cells, excluding those that produce surfactin, exhibit varied responses to it and differentiate into distinct types of specialized cells. The sequential events leading to biofilm formation in *B. subtilis* are driven by the functional relationship between potassium, surfactin, and histidine kinase KinC. Surfactin-induced pores exhibit some selectivity for potassium. The loss of potassium ions is a signal for histidine kinase KinC, a signal transduction protein that responds to potassium leakage by phosphorylating the transcription factor Spo0A. Activated by phosphorylation, Spo0A∼P induces the expression of SinI, which suppresses the repressor SinR. As soon as the SinR repressor ceases its activity, the epsA-O operon, responsible for the synthesis and export of exopolysaccharide, and the three-gene operon yqxM-sipW-tasA, responsible for the protein component of the biofilm matrix TasA, are derepressed and the proteins necessary for biofilm formation begin to be produced (Figure 4).

It is noteworthy that in *Bacillus* members producing surfactin, an evolutionary relationship between surfactin and KinC can be traced. Only the kinC gene and the comQXPA gene cluster were found in them, which is one of the quorum sensing (QS) systems that regulate surfactin production [120]. Surfactin as a signaling molecule was also necessary for biofilm formation among other *Bacillus* species: *B. amyloliquefaciens*, B. velezensis, and *B. licheniformis* [121].

Surfactin-activated Spo0A∼P regulates not only matrix production but also sporulation, cannibalism, and motility. These are the processes that occur in a bacterial population as a response to stressful environmental conditions. In response to stress, *Bacillus* cells increase the concentration of cyclic di-guanosine monophosphate (c-di-GMP), which is a signaling system that plays a key role in inhibiting motility. The action of surfactin on the cell increases the level of Spo0A∼P, which in turn suppresses phosphodiesterase proteins (PDE), which destroy c-di-GMP. As a result, the concentration of c-di-GMP remains high, and c-di-GMP binds to the MotI receptor, which interacts with the flagellar stator element MotA, detaching and isolating it from the flagellar rotor, resulting in loss of motility (Figure 4). This mechanism of motility suppression affects the immotile part of the population, forcing it to remain in its current location, where it forms biofilm and spores. In contrast, the motile cells, which make up the motile portion of the population, enable the transition to new resources and habitats. In this case, Spo0A stimulates the transcription of genes necessary for motility. In addition, surfactin enhances motility, acting as a biosurfactant, reducing the surface tension and blocking the interaction between the substrate and the bacterial cell [25,122,123]. Thus, the surfactin biosynthesis pathway, mediated by the QS system, is linked to regulatory pathways that govern the differentiation of the bacterial population into specialized cells capable of surviving in altered environmental conditions.

## 5. Iturins

Iturins are a family of CLPs containing seven alpha-amino acid residues and one beta-amino acid. Variations in the amino acid structure of iturins cause their significant polymorphism. Members of the iturin family, synthesized by bacteria of the genus *Bacillus*, include iturins, bacillomycins, and mycosubtilin [124]. Iturins are cyclic heptapeptides. They contain an amino acid sequence that forms a ring with a β-amino fatty acid via an amide bond. The heptapeptide has a constant configuration of α-amino acids LDDLLDL, containing invariable D-Tyr 2 and D-Asn 3. The carbon chain of β-amino fatty acid contains from 14 to 17 carbon atoms (Figure 2). Different lengths and isomerism of the fatty acid chain determine the structural heterogeneity of iturins [100,125].

The ComX peptide, which regulates QS, reaches threshold concentrations and triggers sequential phosphorylation reactions of the regulators ComA and DegU. ComA∼P regulates the production of surfactin, and DegU∼P regulates the production of fengycin and iturin. Subpopulations that do not produce the corresponding CLP react to the production of CLP and become other types of specialized cells. Spo0A indirectly regulates the production of CLP; its absence reduces CLP production. ComA∼P, DegU∼P, and Spo0A∼P are the key regulators of cell differentiation processes. Knockout of the iturin genes leads to an increase in ComA∼P levels and cell competence, a decrease in DegU∼P and Spo0A∼P levels, increased motility, reduced biofilm formation ability, and decreased sporulation. The addition of iturins reverses the effects. Surfactin, via Spo0A∼P, regulates the synthesis of biofilm matrix components and blocks flagellar motility. Surfactin regulates the synthesis of biofilm matrix components and inhibits flagellar motility through Spo0A∼P. (Figure 4 was created using the images from Servier Medical Art https://smart.servier.com/, 8 December 2024.)

Most reports on iturin producers and their antagonistic activity against microorganisms focus on the species *B. subtilis* and *B. amyloliquefaciens* and less often on the species *B. velezensis*, *B. licheniformis*, *B. megaterium*, *B. pumilus*, and others [104]. Iturins were first isolated by Delcambe in 1965 from *B. subtilis* [126]. Since then, many iturin-producing strains have been described as *B. subtilis*, but later studies have determined that they belong to other species, in particular to *B. velezensis*, which is part of the phylogenetic clade known as *B. subtilis* group [23].

Iturins exhibit predominantly antifungal activity. The list of fungi susceptible to iturin is extensive, primarily comprising phytopathogens. Thus, iturin A from the *B. subtilis* B-3 strain was active against several species of *Fusarium* and *Penicillium Aspergillus* fungi, and iturin A from the *B. subtilis* KS.03 strain was active against *G. gloeosporioides* [127,128]. The inhibitory effect of iturin from *B. amyloliquefaciens* on fungal strains belonging to the genera *Alternaria*, *Penicillium*, *Botrytis*, and *Fusarium* has been reported, while iturin from *B. velezensis* was active against fungal strains from the genera *Aspergillus*, *Cladosporium*, and *Syncephalastrum* [129,130,131,132]. Iturin produced by the *B. pumilus* HY1 strain exhibited antifungal activity against *A. flavus* and *A. parasiticus*, both of which produce aflatoxins—mutagenic and carcinogenic compounds harmful to animals and humans [133]. Bacillomycin D from *B. vallismortis* was active against fungal phytopathogens of the genera *Fusarium*, *Alternaria*, *Phytophthora* [134]. Bacillomycin L from *B. amyloliquefaciens* showed strong antifungal activity against *Botryis cinerea*, *Penicillium expansum*, *Rhizoctonia solani*, *F. oxysporum* [135]. The action of iturins is not limited to phytopathogenic fungi. It was shown that in an experimental infection of the oral cavity of mice caused by *C. albicans*, iturin A inhibited the growth and adhesion of *C. albicans* cells, as well as the viability of mature biofilms formed by yeast fungus. The observed effect was superior to the effect of nystatin, a drug for the treatment of oral infections caused by *C. albicans* [136]. Mycosubtilin C 17 from the genetically engineered strain *B. subtilis* ATCC 6633 showed strong activity against some species of *Candida*, *Cryptococcus neoformans*, *Saccharomyces cerevisiae*, and *Yarrowia lipolytica* and significantly inhibited the in vitro growth of *F. graminearum*, *F. verticillioides*, and their mycotoxin biosynthesis [76,137]. Some iturins are also active against bacteria. Bacillomycin D, isolated from *B. velezensis* at a low concentration of 2 μg/mL, demonstrated effectiveness against bacteria of the genus *Staphylococcus*, including methicillin-resistant *S. aureus* [69].

The amphiphilic nature of iturins allows them to quickly distribute in phospholipid membranes and interact with them, changing their structure and function. Iturins, penetrating the cell wall, disrupt the integrity of the cell membrane, alter its permeability, and induce pore formation, leading to the loss of intracellular contents, particularly the leakage of ions (Figure 3). As a result of ionic imbalance and disruption of intracellular homeostasis, cell death occurs. Nevertheless, the antifungal effect of iturins significantly exceeds their antibacterial effect, which is attributed to the differences in the composition of fungal and bacterial membranes. The main component of the plasma membrane of fungi is ergosterol. Studies of artificial membranes indicate a strong interaction of iturins with ergosterol and cholesterol and the formation of lipopeptide–sterol complexes, which can contribute to the formation of ion-conducting pores in membranes [120,138]. It has been reported that the alcohol group of sterols may participate in such an interaction, as exemplified by the bacillomycin D–sterol complex [139]. Scanning electron microscopy data provided visual confirmation of damage to the plasma membranes of hyphae and conidia of *F. graminearum* fungi by bacillomycin D, which caused cytoplasmic leakage and plasmolysis [140]. Some possible targets of the antimicrobial action of iturins are not associated with the membranes of fungi and bacteria but with their cell walls, and these targets have been identified in silico. Molecular docking results showed that iturin A from *B. aryabhattai* exhibits affinity for (1,3)-β-D-glucan synthase derived from *C. albicans* and can also bind to the MurA protein, whose functionality is essential for the synthesis of the *Salmonella typhimurium* cell wall [141,142].

The destruction of the cell membrane is not the only mechanism by which iturins suppress the growth and development of fungi. Low concentrations of iturins in the natural habitat of iturin-producing bacteria lead to the death of eukaryotic fungal cells via apoptosis (Figure 3). Recent studies on the antifungal activity of iturin A against *S. cerevisiae* have shown that iturin A activation of voltage-gated potassium (Kv) channels results in K^+^ efflux, which is a prerequisite for initiating cell apoptosis [138]. In addition, the impact of iturins on the fungal cell cycle may be linked to the activation of reactive oxygen species (ROS) production [140,143,144]. It has been shown that the effect of iturin on the phytopathogen *Verticillium dahliae* is associated with the Hog1 mitogen-activated protein kinase (MAPK), which fungi use for intracellular stress signaling, including the osmosensory signaling pathway and regulation of cell cycle processes. Iturin-induced ROS accumulation, Hog1 MAPK activation, and cell wall defects [145]. In the study of the effect of iturin A on *A. niger*, the induction of apoptosis of fungal cells via the oxidative stress pathway was accompanied by an excess of ROS, hyperpolarization of the mitochondrial membrane potential, and accumulation of malondialdehyde [99]. Bacillomycin D from *F. graminearum* induced high ROS accumulation in fungal cells, increasing the expression of specific genes for ROS production and genes for glutathione reductase and thioredoxin, cellular antioxidant enzymes [140].

In addition to the direct inhibitory effect on phytopathogens, iturins are able to interact with host plants and enhance their resistance to infection. In an experiment with the *Arabidopsis* plant, iturin A induced salicylic acid (SA) and JA signaling pathways, through which transcription of the protective genes PR1 and PDF1.2 were activated, respectively (Figure 3). It has been shown that iturin A can bind to several intermediate signaling molecules in the plant host-induced systemic resistance pathway against pathogen infection. Among the common pathways of ISR activation by other CLPs, iturin A has been found to have a specific effect in stimulating ISR. It is realized through the oxylipin biosynthesis system required for the production of jasmonic acid, which is one of the main regulators of ISR [35,146].

Iturins exhibit antiviral activity, including that mediated by the antifusogenic mechanism (Figure 3). The antiviral properties of iturin A and mycosubtilin against SARS-CoV-2, tested in Vero cells, demonstrated a decrease in viral infectivity by 2.0 and 2.5 log, respectively, compared to the control. Pre-incubation of the virus with mycosubtilin and iturin A at a concentration of 12.5 μg/mL and 25 μg/mL prevented the cytopathogenic effect on Vero cells [147]. Molecular docking revealed the interaction of iturin with the ACE2-spike protein as a possible antiviral target [116]. It was also shown in silico that the potential energy of iturin A binding to the nsp12 enzyme, an RNA-dependent RNA polymerase, is comparable to the binding energy of this enzyme to remdesivir triphosphate (RTP), an FDA-approved drug [115]. Iturin A and mycosubtilin also acted as inhibitors of the fusion of the SARS-CoV-2 virus lipid envelope with cell membranes. They showed a pronounced inhibition of POPC/SM/CHOL vesicle fusion, where the fusion trigger was the FP_SARS (816–827)_ viral peptide. At the same time, membrane changes affected lipid packing and curvature stress in lipid bilayers, which led to a decrease in the formation of intermediate fusion structures, and antifusogenic activity rose with an increase in the length of the hydrophobic tail of CLP [147].

The amphiphilic properties of iturins suggest that their primary mechanism of antimicrobial action is associated with cellular membranes, while at the phase boundaries, amphiphilic compounds behave as surfactants, altering the hydrophobic interactions between microorganisms and the surface (Figure 3) [97]. By binding to the surface and changing their physicochemical properties, iturins, like other biosurfactants, prevent microorganism adhesion to the surface. For example, bacillomycin D from *B. amyloliquefaciens* inhibited the biofilm formation of *C. albicans* [134,136].

A decrease in surface tension also enhances the passive motility of CLP-producing bacteria by decreasing adhesion to surfaces. Motility enables organisms to avoid unwanted interactions with competitors and toxic substances while also facilitating the expansion of habitat colonization. Motility function was studied in mutants deficient in any of the iturin compounds. The iturin mutant Jt84 ∆itu showed a slight increase in motility and reduced ability to form biofilms compared to the wild-type *B. velezensis* Jt84 [42]. Bacillomycin L-deficient Δ bac mutants of *B. subtilis* 916 (*B. velezensis 916*) had decreased biofilm formation [148]. It was shown that changes in motility and biofilm formation by iturin producers may depend on their action as signaling molecules. Thus, bacillomycin L acts as an extracellular signal. As a result of the impact on the cell, changes occur in the expression levels of regulatory genes, whose products, in turn, regulate the phosphorylation of key regulators ComA, DegU, and Spo0A, which control competence, motility, biofilm formation, and sporulation (Figure 4) [149]. It was also reported that bacillomycin D acts as a biofilm activator by binding to the KinB-Spo0A-SinI-SinR matrix complex, which triggers biofilm formation [150]. Thus, iturins implement the main mechanisms of antimicrobial action—disruption of membrane integrity and cellular apoptosis, as well as indirect ones—by changing the surface properties, acting as a surfactant, and triggering an immune response in plants to phytopathogens, acting as signaling molecules. On the other hand, iturins are able to trigger their own defense mechanisms against adverse environmental impacts, such as changes in motility and the formation of biofilms.

## 6. Fengycins

Fengycins are cyclic lipopeptides with a complex structure. The peptide part consists of 10 amino acid residues, 8 of which form an internal lactone ring between the amino acid Tyr, located at the third position, and the terminal amino acid Ile. A β-hydroxy fatty acid, typically saturated and with a variable chain length of 14 to 18 carbon atoms, can exist in either linear form or isoform. It is attached to the N-terminal Glu residue at the first position of the peptide chain (Figure 2) [98,100,151]. Fengycins or plipastatins were discovered in 1986 as antifungal antibiotics isolated from the *B. subtilis* F-29-3 strain [152]. Fengycins A and B were initially described as the two main classes of fengycins, differing by one amino acid residue in the sixth position. Since then, several other variants have been identified, all of which contain Glu residues in the first and fifth positions and a Gln residue in the eighth position [151,153].

Among the fengycin producers, there are such species as *B. velezensis*, *B. subtilis*, *B. amyloliquefaciens*, *B. siamensis*, *B. licheniformis*, *B. mojavensis*. Many strains among these species are associated with the rhizosphere of plants and fungal phytopathogens. Indeed, fengycin exhibits predominantly antifungal activity, especially against filamentous fungi such as *F. oxysporum*, *F. solani*, *F. graminearum*, *Alternaria solani*, *Botrytis cinerea*, *Rhozoctonia solani*, *Magnaporthe grisea*, and others [104,154,155,156].

The selective action of fengycins on fungi could not be associated only with the solubilization of the lipid bilayer and the formation of transmembrane pores (Figure 3). Therefore, new data on the interaction between fengycins and fungal membranes have appeared. In complex membrane models prepared from fungal lipids, it was shown that a high content of ergosterol in the membranes reduced the ability of fengycin to bind and integrate into them. In liposomes composed of the same lipids and encapsulated with calcein, fengycin-induced leakage was observed, while membranes with high ergosterol content showed greater resistance to this effect [157]. Aggregation of fengycins in membranes also plays a role in the destruction of fungal cells, which has been established experimentally. The experiment used two membrane compositions: one mimicking a bacterial membrane, consisting of a mixture of palmitoyl-oleoyl-phosphatidylethanolamine (PE) and palmitoyl-oleoyl-phosphatidylglycerol (PG), and another mimicking a eukaryotic membrane, consisting of palmitoyl-oleoyl-phosphatidylcholine (PC). Fengycin was shown to disrupt membranes regardless of lipid composition, with the aggregation propensity changing depending on their composition. Hydrophobic interactions between Tyr-10, Tyr-4, and Ile-11 of adjacent fengycins lead to the formation of stable aggregates, and the aggregation propensity changes depending on the lipid composition. Larger and more stable aggregates were formed in eukaryotic membranes represented by target fungi that were initially susceptible to fengycin [158].

Fengycins exhibit antifungal activity not only by damaging membranes but also by inducing apoptosis of fungal cells. Characteristic apoptotic phenotypes were observed in experiments with different concentrations of fengycin. Suppression of filamentous fungal growth by fengycin at high concentrations (>50 μg/mL) was attributed to membrane structural damage, leading to cell death, which was confirmed by propidium iodide penetration into the mycelium of *Rhizopus stolonifera*. At fengycin concentrations less than 50 μg/mL, apoptosis was observed, accompanied by changes typical of apoptotic cells, such as ROS accumulation, phosphatidylserine externalization, mitochondrial dysfunction, chromatin condensation, and DNA strand fragmentation [159]. Later studies found increased expression of two apoptosis-inducing factors (AIF), AIFM2 and AIF1, in *F. concentricum* treated with lipopeptide from *B. velezensis* FJAT-54560 strain; the presence of these factors may indicate a mitochondria-dependent apoptosis pathway not associated with metacaspase. No changes in metacaspase expression were observed when the fungus was treated with lipopeptide [160]. In general, both metacaspase-dependent and metacaspase-independent apoptosis pathways were found in fungi (Figure 3) [161]. Other researchers were able to show that C 17 fengycin B induces apoptosis of *F. oxysporum* FJAT-31362 in a metacaspase-dependent manner [162].

The antifungal effect of fengycin is realized in another way, where fengycin acts as an inducer of the plant defense response to pathogens. Indeed, fengycin induces systemic resistance in plants to infection caused, for example, by *S. sclerotiorum*, *B. cinerea*, *Plasmopara viticola* [163]. In an in planta experiment, tomato plants treated with fengycin against *S. sclerotiorum* showed increased expression of genes associated with plant defense involved in systemic resistance, including superoxide dismutase (SOD), polyphenol oxidase (PPO), phenylalanine ammonia-lyase (PAL) [164]. In another study, fengycin enhanced plant defense response not only to stress associated with fungal infection but also in its absence. Thus, during the infection of tangerines caused by *P. digitatum*, fengycin triggered the production of chitinase (CHI), which belongs to the group of pathogenesis-related (PR) proteins and provides protection against fungal pathogens. In the absence of *P. digitatum* infection in plants, fengycin induced the expression of key genes in the ET signaling pathway, which triggers ISR [35]. The initiation of ISR depends on the phytohormones ethylene and jasmonic acid, and the accumulation of PR proteins is largely associated with systemic acquired resistance (SAR), which depends on the phytohormone salicylic acid (SA). It is likely that the plant defense response to pathogens induced by fengycin is regulated through various signaling pathways and their interactions with each other. Indeed, the synergism of the JA and SA signaling pathways plays an important role in the interaction between plants and rhizobacteria, and both SAR and ISR responses are dependent on the transcriptional coactivator NONEXPRESSOR OF PR1 (NPR1), a key regulator of plant immunity (Figure 3) [165].

Fengycin has a limited inhibitory effect on bacteria. First of all, fengycin slightly damages bacterial membranes, which was demonstrated on model membranes using POPE/POPG (2:1) mixtures in the presence of fengycin. An increase in membrane disorder and aggregation of fengycin on experimental membranes was observed, but large aggregates were not stable, unlike those on fungal membranes [158]. In some studies confirming the antibacterial action of fengycin, morphological changes on the surface of bacterial cells were found. Indeed, fengycin from the marine strain *Bacillus* sp. YJ17 exhibited antimicrobial activity against foodborne pathogens such as *P. aeruginosa* PAO1, *S. choleraesuis*, and *L. monocytogenes*. After fengycin treatment, the cell wall surface and membranes of *P. aeruginosa* were damaged, resulting in cytoplasmic leakage and cell death. In addition, fengycin-treated bacteria accumulated ROS, which the authors attributed to a decrease in the expression of ROS-scavenging enzymes [166]. In another study, fengycin inhibited three waterborne pathogens *Edwardsiella tarda*, *Vibrio harveyi*, and *Streptococcus anisopliae* [167]. Finally, the effect of fengycin isolated from *B. amyloliquefaciens* on *Xanthomonas axonopodis* pv. *Vesicatoria* and *P. aeruginosa* PA01 were observed using atomic force microscopy (AFM). Topographic changes on the cell surface were observed at low fengycin concentrations, while complete cell damage occurred at high concentrations. While conducting AFM, a decrease in cell size was observed, likely due to the leakage of intracellular contents. The leakage was confirmed by measuring the efflux of potassium ions from fengycin-treated cells. As already mentioned, the ability of fengycin to damage membranes is associated with its aggregation, which in turn depends on the lipid composition of the target cell membrane. It should be noted that the membranes of the bacteria used in this study contain a phospholipid unusual for bacteria—phosphatidylcholine—which could determine their sensitivity to fengycin [168].

Fengycin has antiviral properties, and these properties are determined in the same way as those of surfactins and iturins by the amphiphilic nature of the compounds themselves. Since they have hydrophobic and hydrophilic fragments that can interact with the viral membrane, the antiviral effect of CLP is primarily directed at enveloped viruses. Based on in silico studies, fengycin was predicted to have the potential to kill the SARS-CoV-2 virus. Indeed, the binding energy of fengycin to nsp12, an RNA-dependent RNA polymerase, was comparable to that of the antiviral drugs RTP and STP [115]. In vitro, fengycin was shown to slightly reduce the amount of SARS-CoV-2 viral RNA in infected Vero E6 cells. However, pre-treatment of Vero E6 with fengycin inhibited the binding of SARS-CoV-2 to the cells, which may indicate an interaction between fengycin and the cells. In addition, fengycin inhibited the fusion of the SARS-CoV-2 viral envelope with the host cell membrane (Figure 3) [169]. The same high antifusogenic activity of fengycin was demonstrated on POPC/SM/CHOL vesicles that mimic the fusion of SARS-CoV-2 with cell membranes [147].

Fengycin is an important metabolic product of the host bacterium, and the regulatory pathways of its biosynthesis overlap with pathways that allow bacterial populations to adapt to altered environmental conditions. Biofilm formation increases the resistance of *Bacillus* to environmental stress and allows it to more successfully colonize the plant rhizosphere. It was experimentally established that the regulator of fengycin production, the pleiotropic protein DegQ, also regulates biofilm formation in the *B. subtilis* NCD-2 strain. The degQ-deficient strain showed weak antifungal activity against *B. cinerea* in vitro and reduced biofilm formation compared to the wild-type strain. Notably, disruption of the fengycin synthetase gene did not affect biofilm formation [170]. DegQ is a positive regulator of fengycin production, with the degQ gene activated by the ComP-ComA two-component system from the comQXPA gene cluster encoding the QS system. In turn, ComA positively and indirectly regulates the DegS-DegU system via DegQ, where DegQ activates the phosphoric acid residue transfer from DegS-P to DegU. Phosphorylated DegU-P, which is a response regulator in the two-component DegS/DegU system, regulates the biosynthesis of antimicrobial metabolites by binding to the promoters of their operons, and different levels of DegU-P negatively or positively regulate motility, biofilm formation, and sporulation in cell subpopulations (Figure 4). *Bacillus* strains deficient in comA, degQ, and degU genes significantly reduced fengycin yield, and deletion of degQ and degU genes negatively affected biofilm formation [45,88,170]. ComA is the primary regulator of the response to a cascade of chemical reactions triggered by signaling molecules; therefore, the knockout of *comA* in *B. amyloliquefaciens* leads to a significant decrease in fengycin yield [171]. The processes of fengycin biosynthesis and the biosynthesis of products that lead to changes in motility, biofilm formation, and sporulation have overlapping pathways. In essence, QS regulates key metabolic changes in *Bacillus* in response to environmental shifts, expressed in the differentiation of *Bacillus* cellular subpopulations, and the production of fengycin is closely linked by regulatory mechanisms to these processes [88,170].

## 7. Conclusions

The main CLPs produced by *Bacillus* species are surfactin, iturin, and fengycin, all of which exhibit antimicrobial activity due to their unique physicochemical properties. First, CLPs, as amphiphilic substances, interact with the membranes of microorganisms, causing their damage. The degree of damage can vary, from the formation of channels and pores in the membrane to their disintegration, and depends on the concentration of CLPs, the chemical structure of the compound itself, and the composition of microbial membranes. Together, these three factors determine the predominant membrane damage caused by fengycins and iturins in filamentous fungi, as well as the less specific antimicrobial activity of surfactin. Amphiphilic CLPs, acting as surfactants at interfaces, reduce surface tension and alter hydrophobic interactions between microbial cells and surfaces. In this case, the antimicrobial effect of CLPs is associated with the prevention of microorganism adhesion to the surface and the formation of biofilms. For a long time, CLPs have been considered antimicrobial agents for solving practical problems in biotechnology, medicine, and agriculture, while only a small part of the studies have been devoted to the antimicrobial activity of CLPs in natural conditions. The antimicrobial potential of CLPs in nature is manifested through antagonistic interactions between microorganisms and is realized at lower concentrations than those required in vitro. Low concentrations of CLPs can induce apoptosis in fungal cells, which leads to their death, and also act as inducers of phytohormone signaling pathways, causing a protective reaction of ISR in plants against phytopathogens. Finally, CLPs are important for the host bacterium itself when they regulate the behavior of the population in natural habitats, in particular, the processes of differentiation in response to stressful environmental conditions. In this context, CLPs contribute to the regulation of multicellular behavior within the bacterial population, where subpopulations differentiate into specialized cell types such as competent cells, flagellated cells, matrix-producing cells, and spores. Although the dependence of these processes on CLP has not been sufficiently studied, the presence of common regulatory pathways between the CLP biosynthesis pathways and the pathways that form specialized cells indicates a close connection between CLP and the adaptive mechanisms of the host bacterium.

Indeed, the antimicrobial action of CLP has its own natural purpose, as do the functions of CLP in their own metabolism. These compounds carry essential physiological, ecological, and evolutionary information about their producers, which is valuable not only for discovering new CLPs from *Bacillus* but also for finding and improving known producers of CLPs.

## 8. Prospects

CLPs continue to attract interest from researchers despite the long-standing development of this group of compounds. With the advent of modern molecular genetics and bioinformatics technologies, CLPs are considered candidates for compounds with antimicrobial activity. First, these technologies allow for rapid prediction of CLPs in potential producers and the identification of previously uncharacterized ones. Variants of such compounds can be obtained either by chemical modification or genetic engineering or by total chemical synthesis based on the predicted structure of compounds using the syn-BNP approach. This will likely lead to the emergence of new forms of CLPs, particularly those with reduced cytotoxicity, and to the production of a specific form of a chemical compound. At the same time, additional studies of the functions of CLPs in nature and in the metabolism of host producers will facilitate the discovery of such forms. Finally, given that the development of resistance to CLPs is limited, and the chemical structure of these compounds allows for the production of analogs with improved properties, one can predict an increased interest in CLP research and their use in medicine, pharmaceutics, and agriculture.

## Figures and Tables

**Table 1:** *Bacillus* species and diversity of CLPs.

CLPs	Molecular Formula	Species	Reference
**SURFACTINS**			
**Surfactin** **Glu-Leu-Leu-Val-Asp-Leu-Leu**			
Surfactin (surfactin C)	C_53_H_93_N_7_O_13_	*B. subtilis*	[25,29,30,31,32,33,34,35]
		*B. velezensis*	[4,8,9,10,11,36,37,38,39,40,41,42]
		*B. amyloliquefaciens*	[7,43,44,45,46]
		*B. siamensis*	[47,48,49,50]
		*B. inaquosorum*	[51]
		*B. mojavensis*	[52]
Surfactin A	C_51_H_89_N_7_O_13_	*B. subtilis*	[20]
		*B. velezensis*	[53]
Surfactin B	C_52_H_91_N_7_O_13_	*B. velezensis*	[53]
**Pumilacidin** **Glu-Leu-Leu-Leu-Asp-Leu-Ile**			
Pumilacidin A	C_54_H_95_N_7_O_13_	*B. pumilus*	[54]
Pumilacidin B	C_53_H_93_N_7_O_13_	*B. pumilus*	[54]
Pumilacidin C	C_56_H_99_N_7_O_13_	*B. pumilus*	[54]
**Lichenysin** **Gln-Leu-Leu-Val-Asp-Leu-Ile**			
Lichenysin-G2a	C_51_H_90_N_8_O_12_	*B. licheniformis*	[55]
Lichenysin-G4	C_52_H_92_N_8_O_12_	*B. licheniformis*	[55]
Lichenysin-G6a	C_53_H_94_N_8_O_12_	*B. licheniformis*	[55]
**ITURINS**			
**Iturin A** **Asn-Tyr-Asn-Gln-Pro-Asn-Ser**			
Iturin A	C_48_H_74_N_12_O_14_	*B. velezensis*	[8,9,10,11,56,57,58]
		*B. amyloliquefaciens*	[44,59,60,61]
		*B. siamensis*	[48,49]
Iturin D (A2)	C_48_H_74_N_12_O_14_	*B. safensis*	[62]
		*B. velezensis*	[38]
Iturin A4	C_49_H_76_N_12_O_14_	*B. amyloliquefaciens*	[63]
Iturin A6	C_50_H_78_N_12_O_14_	*B. safensis*	[62]
		*B. amyloliquefaciens*	[63]
Iturin A7	C_50_H_78_N_12_O_14_	*B. amyloliquefaciens*	[63]
Iturin A8	C_51_H_80_N_12_O_14_	*Bacillus* sp. KCB14S006	[64]
		*B. amyloliquefaciens*	[63]
Iturin A9	C_51_H_80_N_12_O_14_	*Bacillus* sp. KCB14S006	[64]
**Iturin C** **Asp-Tyr-Asn-Gln-Pro-Asn-Ser**			
Iturin C	C_48_H_73_N_11_O_15_	*B. subtilis* subgroup	[65]
		*B. velezensis*	[66]
**Bacillomycin D** **Asn-Tyr-Asn-Pro-Glu-Ser-Thr**			
bacillomycin D (Bacillomycin)	C_45_H_68_N_10_O_15_	*B. amyloliquefaciens*	[43,44,45,67,68]
		*B. velezensis*	[9,39,40,41,69,70]
		*B. siamensis*	[47,48]
**Bacillomycin F** **Asn-Tyr-Asn-Gln-Pro-Asn-Thr**			
Bacillomycin F	C_52_H_84_N_12_O_14_	*B. siamensis*	[49]
		*B. inaquosorum*	[51]
**Bacillopeptin** **Asn-Tyr-Asn-Ser-Glu-Ser-Thr**			
Bacillopeptin A	C_46_H_72_N_10_O_16_	*B. methylotrophicus*	[71]
Bacillopeptin B	C_47_H_74_N_10_O_16_	*B. amyloliquefaciens*	[72]
**Mycosubtilin** **Asn-Tyr-Asn-Gln-Pro-Ser-Asn**			
Mycosubtilin	C_55_H_86_N_14_O_16_	*B. subtilis subgroup*	[73,74,75]
		*B. spizizenii*	[76]
**FENGYCINS**			
**Fengycin A Glu-Om-Tyr-Thr-Glu-Ala-Pro-Gln-Tyr-Ile** **Fengycin B Glu-Om-Tyr-Thr-Glu-Val-Pro-Gln-Tyr-Ile**			
Fengycin	C_72_H_110_N_12_O_20_	*B. siamensis*	[50]
		*B. velezensis*	[4,8,9,10,11,41,77]
		*B. subtilis*	[31,78,79]
		*B. amyloliquefaciens*	[7,27,45,46,80]
		*B. inaquosorum*	[51]
		*B. mojavensis*	[52]
**KURSTAKINS**			
**Thr-Gly-Ala-Ser-His-Gln-Gln**			
Kurstakin 1	C_39_H_63_N_11_O_12_	*B. thuringiensis*	[6]
Kurstakin 2,3	C_40_H_65_N_11_O_12_	*B. thuringiensis*	[6]
Kurstakin 4	C_41_H_67_N_11_O_12_	*B. thuringiensis*	[6]
Kurstakin		*B. mojavensis*	[52]

## Data Availability

No new data were created or analyzed in this study. Data sharing is not applicable to this article.
